# Adherence to viral load testing guidelines, barriers, and associated factors among persons living with HIV on ART in Southwestern Uganda: a mixed-methods study

**DOI:** 10.1186/s12889-022-13674-z

**Published:** 2022-06-29

**Authors:** Polly Lubega, Sylivia Juliet Nalugya, Angella Namyalo Kimuli, Majoreen Twinokusiima, Mercy Khasalamwa, Richard Kyomugisa, Jane Kabami, Asiphas Owaraganise

**Affiliations:** 1 Faculty of Medicine, Mbarara University of Science and Technology, Mbarara, Uganda; 2 Department of Nursing, Kabale University School of Medicine, Kabale, Uganda; 3 Infectious Diseases Research Collaboration, Kampala, Uganda; 4 Department of Obstetrics and Gynecology, Mbarara University of Science and Technology, P.O Box 1410, Mbarara, Uganda

**Keywords:** Adherence, Viral load testing, Guidelines, Associated factors, Barriers

## Abstract

**Background:**

Uganda adapted Viral load (VL) testing for monitoring HIV treatment success and virologic failure. However, there is a paucity of data on how the VL testing guidelines are followed in practice in the HIV clinics. This study determined the adherence to national guidelines on VL testing, barriers, and associated factors in persons living with HIV (PLHIV) on ART in southwestern Uganda.

**Methods:**

We conducted a cross-sectional mixed methods study from April to May 2021 at four HIV clinics in southwestern Uganda. Patient chart review using a checklist that captured age, gender, and level of a healthcare facility, dates of ART initiation, dates VL specimens were drawn, line of ART, patient adherence to ART was done. Continuous data were summarized using mean and median and Chi-square was used for categorical data. We performed regression analysis to determine factors associated with adherence to viral load testing guidelines at a 95% level of significance. Key informant interviews with managers of the health facility, ART clinic and laboratory were carried out, and thematic analysis was conducted to explore barriers to adherence to VL testing guidelines.

**Results:**

The participants’ mean (SD) age was 39.9(± 13.1) years, 39.5% were male, 45.8% received care at a general hospital and median duration on ART was 5 years (IQR;3–7). Of the 395 patient charts reviewed, 317 had their VL testing (80.3%) per the guidelines (defined as up to one month post due date). Receiving care at a hospital (aOR = 2.20; 95%CI 1.30–3.70; *p* = 0.002) and increasing patient age (aOR = 1.02; 95%CI 1.02–1.06; *p* = 0.020) were the factors associated with adhering to VL testing guidelines. Long turnaround time of VL results and insufficient VL testing kits were cites by providers as barriers.

**Conclusion:**

We found suboptimal adherence to VL testing guidelines in PLHIV on ART in southwestern Uganda. Increasing patient age and getting care at a higher-level health facility were associated with guideline-based viral VL testing. Long turnaround time of VL test results and inadequate test kits hindered compliance to VL monitoring guidelines. Strategies that target young PLHIV and lower-level health facilities, increase the stock of consumables and shorten VL results turnaround time are needed to improve adherence to VL testing guidelines.

## Background

In an effort to end the human immunodeficiency virus (HIV) pandemic, the World Health Organization (WHO) recommended the initiation of antiretroviral therapy (ART) for all people living with HIV (PLHIV) regardless of their clinical and immunological status [1] and the use of viral load (VL) testing as a gold standard for monitoring HIV treatment success and virologic failure [2]. In 2016, the Uganda national guidelines on prevention and treatment of HIV fully adapted the WHO universal test and treat policy, and the 2020 national guidelines maintained the recommendations [3]. For the 1.5 million PLHIV in Uganda [4], VL testing guidelines recommend baseline VL testing six month after initiating ART and thereafter annually for those aged 20 years; six monthly for those aged 19 years and below once they have achieved viral suppression [5]

There is evidence that regular VL monitoring is a cost effective [6] and is the first choice strategy for identifying poor adherence to ART and detecting treatment failure among PLHIV due to its good specificity and sensitivity [7, 8]. However, many PLHIV on ART are still reporting to healthcare facilities with either no evidence of viral load testing or out-of-schedule results [9, 10]. This potentially delays detection of treatment failure, identification of patients in need of more intensive adherence support, and results in unnecessary switch to expensive and limited 2^nd^ and 3^rd^ line ART regimen options [11].

Delays in VL testing are likely to be related to patient, provider, and system-level factors [12]. Patients may be virally unsuppressed requiring that testing be postponed or miss schedule VL testing appointments. Providers may not be fully aware of the testing guidelines while at system level, disruptions in supplies chain for materials may hinder VL testing. Optimizing the VL testing cascade and compliance to VL testing guidelines requires addressing challenges to VL implementation in resource-poor settings which include 1) poor adherence to WHO and national guidelines on VL monitoring [13], 2) perceived role of VL testing by both clinicians and patients [14], 3) institutional weaknesses in HIV clinic and laboratory, inadequate health facility staff training [12], 4) expensive VL testing consumables and reagents [15], 5) centralized testing and associated delays in relaying results to health facilities [2, 9], and 6) low demand creation at community level [11]. Therefore, after VL testing guidelines have been implemented across the country, it is imperative to study how these guidelines are followed in practice. Moreover, the information on rates of adherence to VL testing guidelines in sub-Saharan Africa is scanty.

This study aimed at finding out the extent to which the national guidelines on VL testing in PLHIV were being implemented at healthcare facilities in southwestern Uganda. The factors associated with guideline-based VL testing, provider perceived barriers to VL testing at the same health facilities were also of interest.

## Methods and materials

### Study design and duration

We conducted a concurrent explanatory mixed methods cross-sectional study using secondary data as a quantitative component and a descriptive qualitative component. The study was conducted as a single snapshot between April and May 2021. However, we abstracted data spanning the period from February 2018 to February 2020 prior to the first COVID-19 lockdown in Uganda which was characterized by significant disruption in patient visit schedules [16, 17].

### Study setting

We conducted the study at four rural HIV clinics in southwestern Uganda. The estimated region’s population is 5 million people [18]. Uganda’s healthcare system operates on a referral system with primary care facilities organized by administrative division from Health Center (HC) II (parish level), HC III (sub-county level) and HC IV (county level), general hospital (district level) to referral hospitals at regional and national levels [13]. Kinoni HCIV, Kabwohe HCIV, Kitagata hospital and Itojo hospital were purposively selected as study sites because of having well-established HIV clinics with high volumes of PLHIV and serving as VL sample collection hubs for the nearby HCs. All sites offer comprehensive HIV prevention and treatment packages at facility or via community outreaches. The services include sensitization on HIV testing and combination prevention options, management of opportunistic infections, nutritional assessment and management of malnutrition, gender-based violence and other sexual reproductive health services like family planning.

Health facility records show that in 2019, the number PLHIV on ART in the HIV clinic were 2,447 at Kitagata hospital, 1,908 at Itojo hospital, 1,300 at Kinoni HCIV and 2,131 at Kabwohe HCIV. The HIV clinic’s clinical care team usually consists of a doctor or clinician who serves as the manager, trained nurses and midwives, laboratory technicians, counsellor, records/data clerk and PLHIV peers. Every PLHIV has a unique clinic identifier assigned at the health center. His/her sociodemographic and clinic data from the treatment card (blue card) are entered into health facility’s electronic medical records (EMR) database. The PLHIV routinely have one clinic visit every 3 months for clinical assessment and drug refills. When the scheduled visit is for VL testing, the date of blood sample collection is documented on the client’s blue card. The drawn VL samples are sent from all HIV clinics to a district laboratory hub for transportation via the National Sample and Results Transport Network to the Central Public Health Laboratories (CPHL) that conducts countrywide VL testing. VL results are returned to the facility and recorded in viral load registers and patient charts (blue cards) [13]. The consolidated guidelines for the prevention and treatment of HIV and AIDS in Uganda recommended frequency of routine viral load monitoring as follows 1) adults—the first VL test should be done 6 months after initiation of ART and thereafter every 12 months, 2) children and adolescents under 19 years of age—the first VL test should be done at 6 and 12 months after initiating ART, 3) HIV positive pregnant and breastfeeding women—at 6 months on ART, repeated every 6 months throughout pregnancy and until cessation of breastfeeding; 4) for non-suppressed individuals—upon completion of intensive adherence counselling; and 5) after every treatment-failure switch—at 6 months [5].

### Study population and eligibility criteria

For the quantitative component, we focused on medical records of PLHIV on ART during the study period, between February 2018 and February 2020. Eligible individuals needed to have been on follow-up care in the same ART clinic for at least 6 months after initiating the ART. For the qualitative component, we interviewed health facility, laboratory, and ART clinic mangers.

### Sample size and sampling procedure

For the quantitative component, a sample size of 420 participants was estimated using the Kish Leslie formula for a single population proportion [19]. We considered a 95% confidence interval, a 5% margin of error, and a conservative 50% proportion given unknown extent of adherence to VL testing in our setting. We factored a 10% non-response rate in the sample size calculation. We analyzed data for 395 participants with complete data on the duration on ART. Systematic random sampling was used to select 105 PLHIV’s records per HIV clinic depending on total number of PLHIV active in care returned by the EMR database query. We divided the number of potential participants by 105 to establish the sampling interval per study site. If the number of potential participants was not divisible by 105, we rounded off the quotient to the nearest whole number. Beginning with a randomly assigned starting clinic ID, we generated the n^th^ ID until 105 participant IDs were generated.

For the qualitative component, we purposively sampled 12 providers by selecting the three focal persons per health facility for the four participating sites, i.e. health facility, HIV clinic and laboratory manager.

### Data collection procedure

This was a mixed method study which applied both quantitative approaches for patient data and qualitative approaches for healthcare providers data. The database used in this study was derived from PLHIV medical records and contained detailed information about demographic and HIV care characteristics. For each PLHIV’s ID selected, required data was extracted from blue card by trained research assistants using the checklist which was designed based on the details captured by the Uganda laboratory requisition forms. The checklist used abstracted age, sex, and level of healthcare facility, dates of ART initiation, dates VL specimens were drawn, line of ART, patient adherence to ART. The ART start date was used to calculate the duration on ART at the time of the VL test.

For the qualitative component, the research assistants trained in qualitative research used an interview guide (supplementary material) designed and pretested based on the predetermined research outcomes to conduct key informant interviews with a purposively selected health facility manager, laboratory lead and ART clinic lead at each study site. Written informed consent for key informants was obtained prior to conducting the interviews which lasted between 40–60 min. Data was recorded using audio recorders and then backed up on an eternal hard drive.

### Study variables

For the quantitative component, the outcome variable was adherence to VL testing guideline defined within one month from the due date. The independent variables were the PLHIV’s age, sex, duration on ART, ART adherence, ART line, VL test indication (routine versus suspected failure), previous VL result and health facility level.

For the qualitative arm, outcomes of interest were emerging themes on barriers to adherence to VL testing guidelines as perceived by healthcare providers.

### Data management and analysis

For quality assurance, research assistants were trained on the essential study documents, data collected with the checklist by the research assistants was reviewed by the supervising investigator and any errors found were rectified. The qualitative interviews were conducted by experienced research assistants. Completed questionnaires were entered into an EPI-Info (www.epidata.dk, version 7.2.1) database and imported into STATA (StataCorp, College Station, Texas, U.S.A) version 15.0 for analysis. Descriptive analysis was conducted on the socio-demographic variables of age, sex, and level of the facility. The extent of adherence to the national VL testing guidelines was calculated as a proportion of total study participants who received VL testing on schedule. Univariable logistic regression was used to determine unadjusted association between sociodemographic, clinical and laboratory variables with the guideline-based viral load testing. All independent variables with *p* < 0.2 were then entered into a multivariable logistic regression model to determine factors independently associated with guideline-based viral load testing. Odds ratios (OR) and 95% confidence intervals (CI) were calculated and *p*-values < 0.05 considered statistically significant in all cases.

For the key informant interviews, the qualitative research assistants transcribed voice data and backed up both the transcripts and audio recordings on a password-protected computer. Thematic content analysis was used to generate themes and subthemes.

### Ethical consideration

Ethical approval was obtained from the Mbarara University Research Ethics Committee (MUST REC); Protocol reference number: 11/02–21. A waiver of consent was obtained to access the required patient records and abstract research data. Written informed consent was obtained from all qualitative study participants.

## Results

### Quantitative component

#### Baseline participant characteristics

Of the 420 charts retrieved, 25 charts had missing data on date of VL testing or ART start and were excluded from analysis. Of the 395 patient charts with complete data, 214 were from HCIV (54.2%) and 181 were from general hospital (45.8%). Majority of the participants were female 60.5% The mean (SD) age was 39.9(13.1) years, and median (IQR) duration on ART was 5(3–7) years as shown in Table1.

**Table 1:** Baseline characteristics of PLHIV at four HIV clinics in southwestern Uganda between 2018 and 2020 (*N* = 395)

Variable		Frequency(percentage)	Mean (± SD) or Median (IQR)
Sex	Male	156(39.5)	
	Female	239(60.5)	
Facility level	HCIV	214(54.2)	
	Hospital	181(45.8)	
Mean age	(years)	395(100)	39.9 ± 13.1
Median duration on ART	(years)	395(100)	5(3–7)

*IQR* Interquartile range

#### Adherence to VL testing guidelines

The VL testing guidelines were adhered to among 317 of the 395 participants, equivalent to 80.3 per cent (95%CI:76.0–83.9), defined up to one month post due date.

#### Factors associated with adherence to VL testing guidelines

Among the PLHIV, the factors associated with guideline-based VL testing were receiving care at a hospital (aOR = 2.20; 95%CI 1.30–3.70; *p* = 0.002) and increasing patient age (aOR = 1.02; 95%CI 1.02–1.06; *p* = 0.020), as shown in Table 2.

**Table 2:** Crude and adjusted odds ratios of factors associated with adherence to VL testing guidelines at four HIV clinics in southwestern Uganda 2018–2020 (*N* = 395)

Factor		VL testing per guidelines *n* = 317	VL testing not per guidelines *n* = 78	Crude OR (95%CI)	*p*-value	Adjusted OR (95%CI)	*p*-value
**Age (years)**				1.02(1.01–1.04)	0.007*	1.02(1.02–1.06)	0.002*
**Sex**	Male	124(39.12)	32(41.01)	Ref		1.0	
	Female	193(60.90)	46(58.09)	0.92(0.55–1.52)	0.750	1.10(0.65–1.87)	0.707
**Facility level**	HC IV	185(58.36)	29(37.18)	Ref		1.0	
	Hospital	132(41.64)	49(62.82)	2.36(1.42–3.94)	0.001*	2.20(1.30–3.70)	0.020*
**ART Adherence**	Good	289(91.17)	76(97.44)	Ref			
	Fair/Bad	28(8.83)	2(2.56)	0.25(0.01–16)	0.790		
**Treatment line**	First line	310(97.79)	74(94.87)	Ref			
	Second line	7(2.21)	4(4.13)	2.56(0.85–0.70)	0.930		
**Ordering indication**	Routine	290(91.48)	76(97.44)	Ref			
	Repeat	27(8.52)	2(2.56)	0.15(0.02–1.19	0.730		
**Previous VL result**	Suppressed	299(94.32)	77(98.72)	Ref			
	Unsuppressed	18(5.68)	1(1.28)	0.28(0.65–1.21)	0.760		
**Duration on ART**	≤ 5 years	194(61.11)	38(48.72)	Ref			
	> 5 years	123(38.89)	40(51.28)	1.78(0.17–3.64)	0.820		

*OR* Odds Ratio, *aOR* Adjusted odds ratio, *CI* Confidence Interval,

^*^p < 0.05

#### Qualitative component

Of the 12 key informants interviewed, 5 were female. By training, 4 were medical doctors, 2 were nurses, 2 were clinical officers, and 4 were laboratory technologists. The average duration in service was 5 years. The provider perceived barriers were:

#### Long turnaround time of VL test results

The providers felt that long turnaround time of results was a barrier to VL testing.

“Sometimes you find that for some patients they(results) have not come. And they(patients) have to wait for subsequent visit find out what were their results”. (Health facility Manger).

“Like now, we just received results of January to February (2021). And they are not for all the orders we made, others are missing or will take months before CPHL resends.” (Manger HIV clinic).

“Like you bleed a client, this is April and you expect the results to come back in May but the results come back in June or July.” (Manager laboratory.

#### Frequent changes of the VL laboratory request forms

Also, the providers felt that unpredictable frequent switch between electronic and printed request forms was a barrier to VL testing.

“…time to time changes in the preferred mode of viral load lab request form have posed difficulty to correct viral load ordering. The shifts between ordering booklets (hard copies), online and phone calls were found to be major sources of confusion at the onsite labs leading to delays and misplacing of patient results”. Manager HIV Clinic.

“Previously, it was inadequate sample collected. But that has since reduced and it is now issues of mismatching the request form with the samples. Sometimes you find a sample is collected, and then the form reaches the CPHL before the sample. So, you find it is rejected from that side”. (Manager laboratory).

#### Inadequate VL testing kits

Furthermore, providers felt that inadequate stocks of VL testing kits was hindering VL testing.

“At times, we get out of stock of kits, viral load kits, for collecting the samples”. (Manager laboratory).

“You can order for a test, and you find that the viral load kits are not in the laboratory.” (HIV Clinic In- charge).

## Discussion

In this mixed-methods cross-sectional study of healthcare providers and PLHIV on ART at HIV clinics in southwestern Uganda, we found suboptimal adherence to VL testing with eight in ten eligible persons being tested per guidelines. Increasing patient’s age and receiving care at the hospital were associated with adherence to VL testing guidelines. Inadequate VL sample collection kits, delay in return of VL results and ambiguities in laboratory request form hindered adherence to VL testing guidelines.

We found lower adherence to VL testing than the 95% target [4]. However, our findings are comparable to what was reported by other studies conducted in sub-Saharan Africa that showed 60% from a population based survey across Uganda [13], 33% central Uganda [10], 54% northern Uganda [20], 32% in South Africa, [21] and 60% in Mozambique [22]. However, our mixed methods study design enabled us to explore the contextual factors responsible for poor VL monitoring. Although, there is an improvement from 20% reported by the WHO survey results in low- and middle-income countries in 2014 [23]. Notably, higher VL testing up to 94% was reported in western Kenya [24], 87% in Eswatini (formerly Swaziland) [25] and 93% in Rwanda [26]. This finding of suboptimal VL testing emphasizes the need for intensified efforts from national governments and partners in resource-limited settings to improve VL monitoring to achieve the UNAIDS 2030 targets. To optimize the VL monitoring cascade, the barriers to implementation of WHO recommended VL testing in resource-poor settings such as 1) nonadherence to VL monitoring guidelines [13], 2) clinicians and patients not perceiving VL testing as critical [14], 3) limited capacity of human resources and supply chain disruptions [12], and 4) low demand creation at community level [11] should be addressed.

### Factors associated with adherence to VL testing guidelines among PLHIV

We found a significant increase in adherence to VL testing guidelines with increasing age of the PLHIV. This agrees with findings of studies in Uganda [27] and South Africa [12] showing that older PLHIV we more likely to receive a VL test relative to younger counterparts. However, studies in Myanmar [28] and Zimbabwe [14] did not find associations between PLHIV’s age and VL testing. Overall, delivery of HIV services to children and youth is difficult across different setting with poorer HIV care cascade outcomes including retention in care and VL suppression rates among children and youth [29]. HIV care service providers need to optimize VL testing and target the children to avert the risk of treatment failure and drug resistance, and countering the need for costlier second-line or third-line ART.

In addition, this study found that PLHIV who received HIV care at hospital were twice likely to receive VL testing as per the guidelines when compared those in care at HCIV. This was consistent with studies done in Zimbabwe [14] and Uganda [20] reporting better VL monitoring at hospitals than clinics. Although, Asio and colleagues found no statistically significant difference in the performance of VL monitoring across Ugandan HIV clinics, by level and ownership, better adherence was noted regional referral hospital [13]. The HIV clinics at hospitals when compared to those at level IV or clinics are likely to have better capacity in terms of trained healthcare workforce and access to resources required for VL testing.

### Provider perceived barriers to adherence to VL testing guidelines among PLHIV on ART

Healthcare workers perceived inadequate VL testing consumables and long turnaround time of VL test results as barriers to adherence to guidelines. Optimally, turnaround time should not exceed weeks from sample draw [30]. Similar to our study, healthcare providers in Malawi [14], Lesotho [9] and Cameroon [31] felt that delayed return of VL results was a barrier to VL testing. Additionally, Teri and colleagues [32] also noted that logistical challenges and equipment limitations as barriers to VL testing in resource-poor setting. The overarching concern is the negative effect these barriers pose to the effective utilization of VL results for clinical decision making. We think that irrespective of the nature of barriers to VL testing, it is crucial to first identify them in areas where they occur so that context-specific interventions are designed and executed. Moreover, in low-income countries, adherence to HIV care treatment and monitoring guidelines are generally limited by poorly funded and fragile healthcare systems.

More broadly, our study shows the challenges to VL implementation in resource-poor settings associated with poor adherence to the national VL testing guidelines. It is possible that providers may not conduct VL testing on patients who attend their scheduled visits depending on their perception of available consumables. To optimize the VL testing cascade and improving compliance, and achieve the UNAIDS 2030 last “95”, these gaps must be addressed.

### Strengths and limitations

The strengths of our mixed-methods cross-sectional study include random sampling of patient charts and exploring the provider perceptions that reflect the real-world situation of viral load monitoring in rural Uganda. Our study has some limitations. We retrospectively analyzed secondary programmatic data with notable missingness for data on VL testing dates (6%). Nevertheless, this did not influence study findings. Also, the study was conducted in one region of Uganda which limits generalizability. But, we believe the findings can be extrapolated to other low-income and rural settings.

## Conclusion

We found that adherence to VL testing guidelines was achieved for eighty percent of PLHIV attending HIV clinics in southwestern Uganda. Adherence to VL testing guidelines improved with increasing patient age and receiving HIV care at hospital-level health facility. Adherence to VL testing guidelines was hindered by ambiguities in VL ordering tools and delayed return of test results. Strategies are needed to improve adherence to VL testing including focus on lower-level health centers and younger patients, intuitive VL monitoring tools and adequate sample collection supplies, and point of care testing to shorten results turnaround time.

## Data Availability

De-identified data sufficient to produce primary study findings will be made available on reasonable request to Faculty of Medicine, Mbarara University of Science and Technology. Data requests can be submitted through the corresponding author.

## Abbreviations

ART: Antiretroviral therapy
CPHL: Central Public Health Laboratories
EMR: Electronic medical records
HC: Health Center
HIV: Human immunodeficiency virus
MUST REC: Mbarara University Research Ethics Committee
PLHIV: People living with HIV
VL: Viral load
WHO: World Health Organization
